# Volume kinetics of crystalloid and colloid solutions administered to healthy anesthetized cats

**DOI:** 10.1371/journal.pone.0333135

**Published:** 2025-09-22

**Authors:** Chien-Hsien Kitty Yang, Xiu Ting Yiew, Robert G. Hahn, William Muir, Carolyn Kerr, Shane Bateman

**Affiliations:** 1 Department of Clinical Studies, Ontario Veterinary College, University of Guelph, Guelph, Ontario, Canada; 2 Karolinska Institutet Danderyds Hospital (KIDS), Stockholm, Sweden; 3 Basic Sciences and Research, Lincoln Memorial University, Harrogate, Tennessee, United States of America; Akita Cerebrospinal and Cardiovascular Center, Research Institute for Brain and Blood Vessels, JAPAN

## Abstract

This prospective experimental study evaluated the disposition of a crystalloid and a colloid solution in 10 healthy cats under general anesthesia. Each cat was randomly assigned to receive either 20 mL/kg of a balanced isotonic crystalloid solution (PLA) or 5 mL/kg of 6% tetrastarch 130/0.4 solution (T-HES), administered over 15 minutes, in a 2-period, 2-treatment crossover design. Blood samples were collected, and urine output was measured during a 3-hour experimental period. Plasma dilution was calculated using serial hemoglobin concentrations and red blood cell count. Volume kinetics (distribution and elimination) of each fluid were determined using non-linear mixed effects pharmacokinetic modeling software. Data from a previous study with a similar methodology in healthy conscious cats were included in the population kinetic analysis, revealing anesthesia as a significant covariate for *k*_21_ (peripheral-to-central intercompartmental rate constant) for PLA and *k*_10_ (dilution-dependent first-order elimination rate constant) for T-HES. Cumulative urine output under general anesthesia was approximately 3.5 times lower for PLA and 2.5 times lower for T-HES compared to conscious cats. Overall, our data suggest that the elimination of PLA and T-HES is markedly reduced, and a bolus of PLA produces a short period of plasma expansion with the potential to cause significant peripheral fluid accumulation in cats during general anesthesia.

## Introduction

Fluid therapy is a standard of care for numerous diseases across multiple disciplines, aiming to preserve effective circulating volume, maintain systemic tissue perfusion, and ensure adequate tissue oxygen delivery [1]. These goals can be achieved by infusing a variety of crystalloid or colloid solutions that initially expand the plasma volume. Intravenous (IV) fluid therapy improves outcomes when administered to fluid-responsive patients [2]; however, excessive administration or use in fluid-nonresponsive patients can lead to fluid overload, edema, and tissue hypoperfusion [3]. The veterinary literature remains comparatively devoid of objective, randomized controlled trials (evidence-based research) investigating fluid disposition, especially in the feline species [4,5]. Anecdotal evidence suggests that cats are particularly susceptible to fluid overload. A case-controlled study reported an overall risk of 0.24% for anesthetic- and sedation-related death in cats, with fluid therapy associated with a four-fold increase in the odds of death after adjusting for health status, age, and the presenting procedure [6].

A group of Swedish researchers have adapted pharmacokinetic principles to evaluate the dilutional effect of IV fluids in humans (i.e., “volume kinetics”: VK) [7]. This innovative approach allows IV fluids to be studied similar to pharmaceutical drugs, thereby providing insights into their disposition (i.e., distribution, elimination) under various physiologic and pathologic conditions [7–9]. Volume kinetic models for IV fluid disposition have been proposed for humans and refined over the past 25 years; however, only a few experimental studies have been conducted in animals [8–12]. A 2017 pilot VK study in healthy conscious cats demonstrated the feasibility of VK analysis in this species but highlighted distinct differences from humans [8]. General anesthesia significantly impacts crystalloid VK parameters in humans [13–15], sheep [11]; however, whether these results can be extended to cats remains undetermined. A retrospective VK study in anesthetized humans found that anesthesia-induced decrease in mean arterial pressure (MAP) from 110 to 60 mmHg resulted in a 90% decrease in *k*_10_ [15]. The primary objective of this study was to evaluate and describe the VK of an iso-osmotic balanced crystalloid and a colloid solution in healthy cats during general anesthesia [8]. We hypothesized that anesthetized cats would demonstrate rapid fluid distribution to a peripheral fluid space, along with significantly slower fluid elimination. Additionally, we hypothesized that MAP would be a significant factor (i.e., covariate) for fluid elimination for both fluids.

## Materials and methods

### Animals

The protocol was approved by the Animal Care Committee at the University of Guelph and conducted in accordance with its guidelines from June to July 2020. Purpose-bred cats were obtained for this study thus no written or verbal consent was required. Ten healthy, intact male domestic shorthair cats (median age: 12 months, range 11 to 16.5 months; mean body weight of 4.64 ± 0.46 kg) were included in the study. All cats were deemed healthy based on physical examination and the results from a complete blood count, biochemistry profile, urinalysis, symmetric dimethylarginine level, total thyroxine level, N-terminal pro-brain natriuretic peptide concentration, and feline leukemia virus and feline immunodeficiency virus rapid immunoassay. The cats underwent a 7-day acclimatization period before the experiment. All cats were group-housed in a 12-hour light-dark, temperature-controlled facility and were provided commercial dry cat food and water *ad libitum*.

### Design and treatment

This was a prospective, randomized, blinded, crossover experimental study. Each cat was randomly assigned to receive either 20 mL/kg of a balanced isotonic crystalloid solution (Plasma-Lyte A Injection, Baxter Corporations, Mississauga, ON, Canada; PLA) or 5 mL/kg of 6% tetrastarch 130/0.4 solution (Voluven®, Fresenius Kabi Canada Ltd., Toronto, ON, Canada; T-HES) intravenously over 15 minutes in a 2-period, 2-treatment crossover design. Fluid doses were calculated to achieve approximately equipotent plasma volume expansion [8]. Block randomization was used to achieve equal treatment groups, and each cat received both treatments on separate days, with a minimum 72-hour washout period. Room temperature IV fluids were delivered via a cephalic IV catheter using a fluid pump (Vet-Pro VIP 2000 Veterinary Infusion Pump, Caesarea Medical Electronics Ltd., Staufenburgstr, Lichtenstein, Germany). An independent collaborator randomized the treatment order and managed fluid delivery to maintain investigator blinding.

### Instrumentation

A central venous jugular catheter (Pediatric Two-Lumen Central Venous Catheterization Kit with Blue FlexTip® Catheter, 4 Fr. by 13 cm, Arrow International, Inc., Reading, PA, USA.) was placed in each cat under general anesthesia at least two days before the experiment. Catheters were anticoagulated daily with 100 U/mL of unfractionated heparin (Heparin Sodium Injection USP, Fresenius Kabi Canada Ltd., Toronto, ON, Canada), and insertion sites were inspected daily. If sampling difficulties persisted despite flushing with 0.9% normal saline, the affected catheter lumen was treated with 1 mg/mL of tissue plasminogen activator (Activase® rt-PA, Hoffmann-La Roche Limited, Mississauga, ON, Canada).

Food was withheld for 12 hours before each experiment. Just prior to anesthesia, hydromorphone 0.05 mg/kg IV (Hydromorphone HCl Injection, Baxter Corporation, Mississauga, Ontario, Canada) and midazolam 0.3 mg/kg IV (Midazolam Injection, Fresenius Kabi Canada Ltd., Toronto, Ontario, Canada) were administered via the central venous catheter. General anesthesia was induced with 2–4 mg/kg of propofol IV, administered to effect. The cats were orotracheally intubated and connected to a Bain non-rebreathing circuit. Anesthesia was maintained with isoflurane in oxygen. Mechanical ventilation was initiated using a volume-cycled, pressure-regulated ventilator (Hallowell EMC Model 2000, Hallowell, Pittsfield, MA, USA) with a tidal volume of 10–15 mL/kg (maximum pressure: 20 cmH_2_O) at a rate of 8–12 breaths/min, targeting an end-tidal carbon dioxide (EtCO_2_) of 30–35 mmHg. A 22-gauge, 1-inch IV catheter was placed in the cephalic vein. A polytetrafluorethylene urinary catheter (Slippery Sam Tomcat Urethral Catheter, Smiths Medical ASD Inc., St. Paul, Minnesota, USA) was aseptically advanced into the bladder via the urethra, and a closed system was created by attaching a 50 mL syringe to facilitate urine quantification.

Cats were positioned in sternal recumbency. Blood pressure was monitored using a Doppler sphygmomanometer (811-B Ultrasonic Doppler Flow Detector, Parks Medical Electronics Inc., Aloha, OR, USA) and an oscillometric sphygmomanometer (Cardell® Veterinary Vital Signs Monitor, Model 9401, Midmark, Tampa, FL, USA) with the cuff placed on the antebrachium. Electrocardiogram, EtCO_2_, end-tidal isoflurane (EtISO), and hemoglobin oxygen saturation were continuously recorded by a multiparameter monitor (Daytex Ohmeda S5, Datex Ohmeda Inc., Madison, WI, USA). Anesthetic depth was assessed based on palpebral response and jaw tone. Circulating warm water and warm air blankets were used to maintain body temperature between 37°C and 39°C. Following the 3-hour data collection period, all cats were allowed to recover from anesthesia.

Following instrumentation, a bolus of 40 mg/kg exogenous creatinine (Powered Exogenous Creatinine Anhydrous, Spectrum Chemical Mfg. Corp., New Brunswick, NJ, USA) mixed in 2.5 mL of sterile water (Sterile water USP, Hospira Inc., Lake Forest, IL, USA) was administered intravenously as part of a separate study investigating glomerular filtration rate. To ensure equilibrium and distribution of creatinine, at least 60 minutes were allowed to pass before the VK experiment, wherein baseline blood samples were collected, and a fluid bolus was administered.

### Measurements and data collection

**Fig 1:**
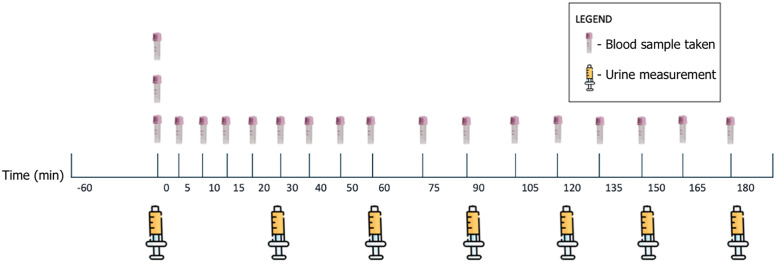
Volume kinetics experimental timeline illustrating blood sampling and urine volume measurements in 10 healthy anesthetized cats.

Triplicate baseline blood samples (0.5 mL x 3) were collected from the central venous catheter using the push-pull technique immediately before initiating the IV fluid bolus infusion (time 0) [16,17]. Single blood samples (0.5 mL) were collected every 5 minutes for the first 20 minutes, every 10 minutes until the 60-minute point, then every 15 minutes for the remainder of the 3-hour experimental period (19 blood samples including triplicate baseline samples) for VK analysis (Fig 1). The catheter lumen was flushed with 0.5 mL of saline after each collection. Hemoglobin (Hb) concentration, red blood cell (RBC) count, and hematocrit (HCT) were measured using an automated hematology analyzer (Siemens ADVIA 2120i Hematology Analyzer, Siemens Healthcare GmbH, Henkestr, Erlangen, Germany). Cardiorespiratory parameters were recorded at each blood sampling time point. Bladder volume was measured at baseline and every 30 minutes for 3 hours by emptying the bladder via the closed urinary catheter system, with complete emptying confirmed by ultrasound. Urine volume was measured using the attached sterile syringe then returned to the bladder after measurement. The bladder was fully emptied, and the urine was discarded at the end of each experiment.

### Plasma dilution

The mean of the three baseline Hb and RBC measurements was used for each experiment’s initial baseline value. Mean plasma dilution at each time point was determined from serial Hb concentrations and RBC measurements using the following equation (Microsoft Excel®, Microsoft Canada, Toronto, ON, Canada):


$$\text{Plasma}\;\text{dilution},\;\;\frac{\left(v-V\right)}{V}=\left[\frac{Hb/{Hb}_{\left(t\right)}-\text{1}}{\text{1}-HCT}+\;\frac{RBC/{RBC}_{\left(t\right)}-\text{1}}{\text{1}-HCT}\right]÷\text{2}$$

The symbols in the equation are defined as such: *v*, volume of distribution of IV fluid; *V*, volume of expandable body fluid space; *v*-*V*, absolute volume expansion, $\frac{\left(v-V\right)}{V}$, fractional plasma dilution; *Hb*, hemoglobin concentration; *HCT*, hematocrit; *RBC*, red blood cell count

Correction for plasma dilution may be necessary when larger blood volumes are sampled during VK studies [18]. A preliminary analysis comparing corrected and uncorrected data showed that such correction had minimal impact on the kinetic output. Thus, the remaining VK analyses were performed using the uncorrected mean Hb-RBC plasma dilution.

### Statistics

Descriptive data analysis was performed (RStudio Open Source Edition. RStudio: Integrated Development for R. RStudio, PBC, Boston, MA, USA). Oscillometric systolic (SAP), diastolic (DAP), mean (MAP), and Doppler (DBP) derived blood pressures, along with heart rate (HR), were not normally distributed (Shapiro-Wilk test) and reported as median and interquartile range (IQR). Body weight, urine output, and volume of fluid administered were normally distributed and reported as mean ± standard deviation (SD). Volume kinetic analysis was performed using non-linear mixed-effects pharmacokinetic modeling and simulation software (Phoenix® NLME^TM^ version 8.3, Certara, St. Louis, MO, USA). The VK model parameter estimates are reported as mean and 95% confidence interval (CI).

Volume kinetic modeling was performed on data obtained from the current experiment (S1 File); however, a stable model with fixed parameters (base model) was not achieved, precluding covariate analysis. Data from the current study were analyzed alongside data from a previous study in healthy conscious cats [8] to perform population VK and covariate analyses. The descriptive data and hemodynamic parameters of the anesthetized cats were compared with the conscious cats using two sample T-test and Mann-Whitney U test, and considered significant if p-value was < 0.05.

### Volume kinetic modeling

Population maximum likelihood modeling with microconstant parameterization was performed using the Naïve Pooled algorithm, optimized with the First-Order Conditional Estimation with Extended Least Squares (FOCE-ELS) algorithm. A one-volume fluid space (1-VOFS) and two-volume fluid space (2-VOFS) kinetic models were fitted separately for each fluid type. Plasma dilution $\left(\frac{\left(v-V\right)}{V}\right)$, intravenous infusion rate (*R*_0_), time, and subject ID were the input variables used by the models to generate the best parameter estimates (*V*, *k*_10_ in 1-VOFS; *V*_*c*_, *k*_10_, *k*_12_*, k*_21_ in 2-VOFS). Cumulative urine output was included as an input variable for the PLA group but not for the T-HES group due to its complex elimination that is not entirely accounted for through urinary excretion.

The differential equation used to describe the kinetic models were:


$$\text{1}\text{-}\;\text{VOFS}\;\;\frac{dv}{dt}=R_{\text{0}}-\;k_{\text{1}\text{0}}(v-V)$$

$$\text{2}\text{-}\;\text{VOFS}\;\;\frac{dv_{c}}{dt}=\;R_{\text{0}}-\;k_{\text{1}\text{0}}\left(v_{c}-V_{c}\right)-\;k_{\text{1}\text{2}}\left(v_{c}-V_{c}\right)+\;k_{\text{2}\text{1}}(v_{p}-V_{p})$$

$$\frac{dv_{p}}{dt}=\;k_{\text{1}\text{2}}\left(v_{c}-V_{c}\right)-k_{\text{2}\text{1}}(v_{p}-V_{p})$$

The statistical fit of the 1-VOFS and 2-VOFS kinetic models was compared, and the base model for subsequent covariate analysis was selected based on the lowest Akaike Information Criterion (AIC) value, provided the parameter estimates were physiologically plausible (relative to other species) and had coefficients of variation (CV%) < 50%.

### Covariate analyses

Potential covariates in the VK model were identified from the correlation ratio (eta, η)-covariate box and scatter plots of body weight, use of general anesthesia, and time-varying hemodynamic parameters. Covariate analysis was performed manually and verified using the stepwise covariate search function (forward addition, backwards elimination), with thresholds for adding or removing a covariate effect set at p = 0.05 and p = 0.01, respectively. A covariate effect was added to the model if it reduced the −2 Log Likelihood (−2LL) by more than the critical value of 3.84 (p < 0.05) and removed if it increased the −2LL by less than the critical value of 6.64 (p < 0.01). A covariate effect was considered statistically significant if the 95% CI did not include 0.

### Plasma volume expansion and fluid simulation

Volume kinetic parameter estimates generated from the final models after incorporating significant covariate effects were used to illustrate PLA and T-HES fluid kinetics and plasma volume expansion visually using the Phoenix^®^ NLME^TM^ simulation function.

## Results

All ten cats completed the study. One cat required the replacement of a central venous catheter due to inadvertent dislodgement, and two cats had catheter securing sutures replaced. Tissue plasminogen activator successfully restored flow in one catheter. Mild pyoderma developed at the central venous catheter insertion site in one cat; the site was cleaned thoroughly and treated for 7 days with oral antibiotics (Clavamox® Drops, Zoetis Canada Inc., Kirkland, QC, Canada).

A total of 20 data sets (10 PLA; 10 T-HES) were acquired from 10 anesthetized cats. During each experiment, 28 blood samples (~14 mL of whole blood) were collected from each cat for the current VK study and concurrent studies investigating glomerular filtration rate and endothelial glycocalyx. Each data set included 17 serial Hb and RBC measurements, resulting in 340 measurements, three of which were excluded from analysis due to missing data. The mean fluid volumes administered were 92.8 ± 8.95 mL for PLA (20 mL/kg) and 23.2 ± 2.49 mL for T-HES (5 mL/kg). The mean cumulative urine output over the 180-minute (3-hour) experimental period was 10.13 ± 3.17 mL (0.73 ± 0.23 mL/kg/hr) for PLA and 7.12 ± 2.12 mL (0.51 ± 0.15 mL/kg/hr) for T-HES. Cumulative urine output accounted for 11% and 31% of the infused PLA and T-HES volumes, respectively.

**Fig 2:**
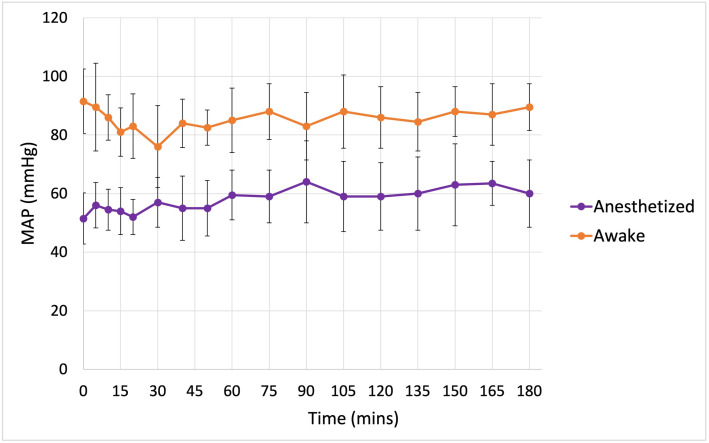
Median mean arterial pressure in 10 healthy male anesthetized cats (20 experiments) and 10 conscious cats (19 experiments) receiving two fluid types. The interquartile range is displayed on the plot.

Data sets from a previous study [8], including 9 from the PLA group and 10 from the T-HES group, were incorporated into the population VK analysis (Table 1). These data were obtained from 10 healthy, conscious cats that received the same PLA (20 mL/kg) and T-HES (5 mL/kg) doses as in the current study. The mean cumulative urine output over the 180-minute experimental period in the conscious cats, estimated by ultrasonography, was 38.74 ± 27.63 mL (2.68 ± 1.91 mL/kg/hr) for PLA and 19.55 ± 14.85 mL (1.36 ± 1.03 mL/kg/hr) for T-HES, representing 40% and 81% of the infused PLA and T-HES volumes, respectively [8]. The overall median MAP of anesthetized and awake cats is significantly different (p < 0.001), the trend of MAP over the experimental period of anesthetized and conscious cats are shown in Fig 2.

**Table 1:** Descriptive statistics of patient characteristics and hemodynamic parameters in 20 healthy male cats receiving two fluid types under general anesthesia (E = 20) compared to those when conscious (E = 19) [8].

Parameter	Anesthetized Cats (E = 20)	Conscious Cats (E = 19) [8]	p-value
Body Weight^a^	4.64 ± 0.46 kg	4.81 ± 0.69 kg	0.358^§^
Age^b^	12 (11.0–16.5) months	8 (6.9–8.4) months	< 0.001^¶^
Cumulative Urine Output^a^	PLA (n = 10) 10.13 ± 3.17 mL 0.73 ± 0.23 mL/kg/hr	PLA (n = 9) 38.74 ± 27.63 mL 2.68 ± 1.91 mL/kg/hr	0.015^§^
	T-HES (n = 10) 7.12 ± 2.12 mL 0.51 ± 0.15 mL/kg/hr	T-HES (n = 10) 19.55 ± 14.85 mL 1.36 ± 1.03 mL/kg/hr	0.003^¶^
HR^b^	156 (143–169) bpm	163 (140–195) bpm	< 0.001^¶^
SAP^b^	96 (84–107) mmHg	117 (109–125) mmHg	< 0.001^¶^
MAP^b^	57 (50–70) mmHg	86 (75–94) mmHg	< 0.001^¶^
DAP^b^	35 (30–42) mmHg	56 (42–73) mmHg	< 0.001^¶^
DBP^b^	84 (68–103) mmHg	Not measured	

n, number of animals; E, number of experiments; HR, heart rate; SAP, oscillometric systolic blood pressure; MAP, oscillometric mean arterial blood pressure; DAP, oscillometric diastolic blood pressure; DBP, Doppler blood pressure, bpm; beats per minute; PLA, Plasma-Lyte A; T-HES, 6% tetrastarch 130/0.4.

^a^value reported as mean ± standard deviation.

^b^Value reported as median (interquartile range).

§ Two sample T-test, statistical significance defined as p < 0.05.

¶ Mann-Whitney U test, statistical significance defined as p < 0.05.

### Population VK analysis for conscious and anesthetized cats

**Fig 3:**
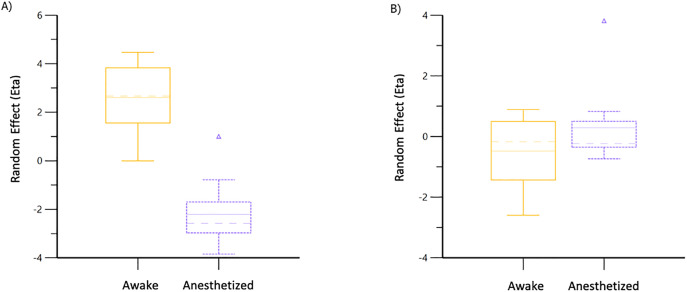
Population covariate boxplot for *k*_21_ in A) Combined PLA base model, B) Combined PLA final model. The random effect on *k*_21_ was accounted for by including anesthesia in the model.

**Fig 4:**
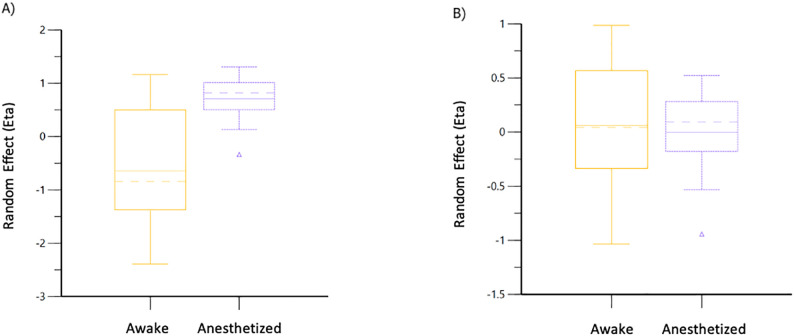
Population covariate boxplot for *k*_10_ in A) Combined T-HES base model, B) Combined T-HES final model. The random effect on *k*_10_ was accounted for by including anesthesia in the model.

Volume kinetic data from anesthetized cats are provided as supplementary files (S1 File). These data were then combined with those from conscious cats [8]. The combined data sets for PLA (E = 19) and T-HES (E = 20) were analyzed separately by fluid type, and 1-VOFS and 2-VOFS kinetic models were created. The 1-VOFS kinetic model provided a statistically justified fit for T-HES, while the 2-VOFS kinetic model was justified for PLA. Covariate analysis revealed that anesthesia produced a significant effect on *k*_21_ for PLA (Fig 3) and *k*_10_ for T-HES (Fig 4). Although heart rate was identified as a covariate for *k*_21_ in cats receiving PLA, it was not considered significant because the 95% CI included zero. The covariate effects of DBP, EtISO, EtCO_2_, and temperature could not be determined since these data were unavailable from the previous study in conscious cats [8].

For PLA group: $k_{\text{2}\text{1}\;anesthetized\;cats}=\;k_{\text{2}\text{1}\;awake\;cats}e^{-\text{6}.\text{0}\text{2}\text{8}}$
For T-HES group: $k_{\text{1}\text{0}\;anesthetized\;cats}=\;k_{\text{1}\text{0}\;awake\;cats}e^{\text{1}.\text{8}\text{2}\text{7}}$

**Fig 5:**
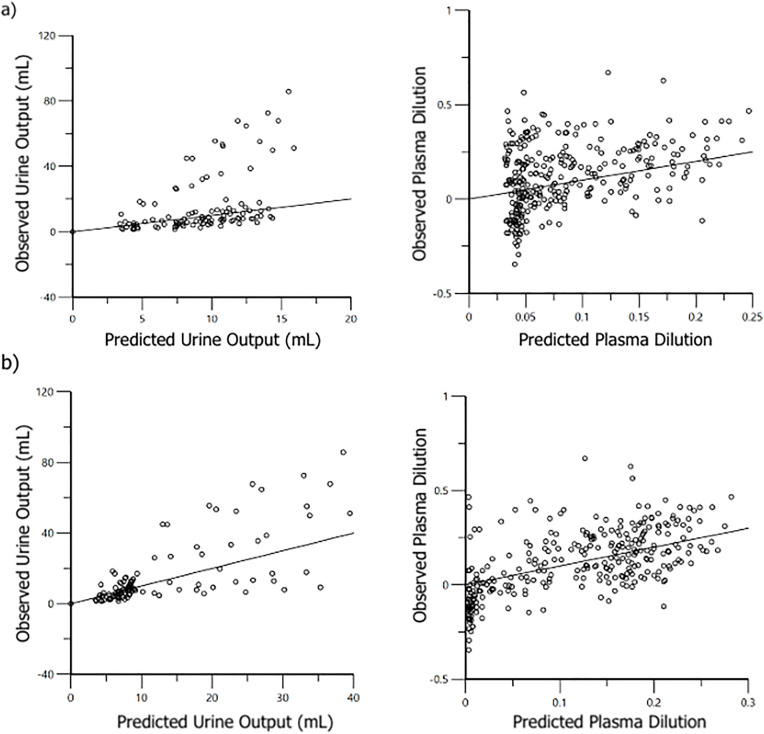
Population residual plots for the two-volume of fluid space model of Plasma-Lyte-A (PLA) in healthy anesthetized (n = 10) and conscious (n = 9) male cats. Ideally, data points should fall close to the line of unity (y = x). a) Base model without covariates. b) Final model incorporating anesthesia as a significant covariate on *k*_21_.

**Fig 6:**
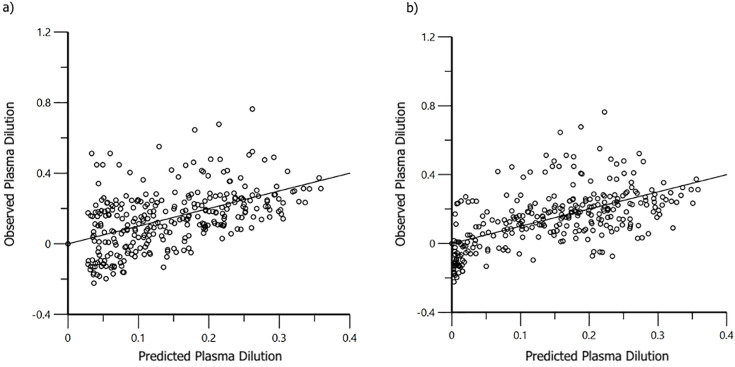
Population residual plots for the one-volume of fluid space model of 6% tetrastarch 130/0.4 (T-HES) in healthy anesthetized (n = 10) and conscious (n = 10) male cats. Ideally, data points should fall close to the line of unity (y = x). a) Base model without covariates. b) Final model incorporating anesthesia as a significant covariate on *k*_10_).

Model-predicted urine output and plasma dilution demonstrated an improved fit with observed values for PLA and T-HES after accounting for general anesthesia as a covariate (Figs 5 and 6). The VK parameter estimates for the final PLA and T-HES models, with and without covariate analysis, are reported in Table 2. Final VK parameter estimates, adjusted for significant covariance effects, for anesthetized and conscious cats are reported in Table 3.

**Table 2:** Population volume kinetic parameter estimates for combined datasets obtained from healthy anesthetized and conscious male cats.

Fluid type	VK model	VK parameter	Estimate mean (95% CI)	CV (%)	−2LL
PLA (E = 19)	2-VOFS	*V*	372.80 mL (233.00–512.61)	19.1	184.08
		*k*_10_	0.0028/min (0.0019–0.0037)	15.6	
		*k*_12_	0.0296/min (0.0201–0.0390)	16.3	
		*k*_21_	0.0070/min (0.0022–0.0118)	34.5	
	2-VOFS with significant covariate	*V*	355.82 mL (217.87–493.79)	19.7	166.48
		*k*_10_	0.0029/min (0.0019–0.0039)	17.3	
		*k*_12_	0.0319/min (0.0220–0.0419)	15.8	
		*k*_21_	0.1608/min (−0.082–0.404)	76.8	
		Effect of anesthesia on *k*_21_ (covariance)	−6.028 (−9.492 to −2.564)	−29.2	
T-HES (E = 20)	1-VOFS	*V*	73.01 mL (61.07–84.95)	8.3	−538.03
		*k*_10_	0.0128/min (0.0067–0.0189)	24.3	
	1-VOFS with significant covariate	*V*	77.85 mL (63.41–92.28)	9.4	−551.83
		*k*_10_	0.0043/min (0.0016–0.0070)	31.6	
		Effect of anesthesia on *k*_10_ (covariance)	1.827 (1.16–2.49)	18.5	

E, number of experiments; CI, confidence interval; CV, coefficient of variation, −2LL, −2 Log Likelihood; PLA, Plasma-Lyte A; T-HES, 6% tetrastarch 130/0.4; VK, volume kinetics; 1-VOFS, one-volume of fluid space; 2-VOFS, two-volume of fluid space; *V*, volume of expandable body fluid space, *k*_10_, first-order elimination rate constant; *k*_12_, central to peripheral intercompartmental rate constant; *k*_21_, peripheral to central intercompartmental rate constant.

**Table 3:** Final volume kinetic parameter estimates, adjusted for significant covariates, for healthy anesthetized and conscious male cats.

Fluid Type	VK Parameter	Anesthetized Cats	Conscious Cats
PLA (E = 19)	*V*	355.82 mL	355.82 mL
	*k*_10_	0.0029/min	0.0029/min
	*k*_12_	0.0319/min	0.0319/min
	*k*_21_	0.0004/min	0.1608/min
T-HES (E* *= 20)	*V*	77.85 mL	77.85 mL
	*k*_10_	0.0267/min	0.0043/min

E, number of experiments; PLA, Plasma-Lyte A; T-HES, 6% tetrastarch 130/0.4; VK, volume kinetics; *V*, volume of expandable body fluid space, *k*_10_, first-order elimination rate constant; *k*_12_, central to peripheral intercompartmental rate constant; *k*_21_, peripheral to central intercompartmental rate constant.

**Fig 7:**
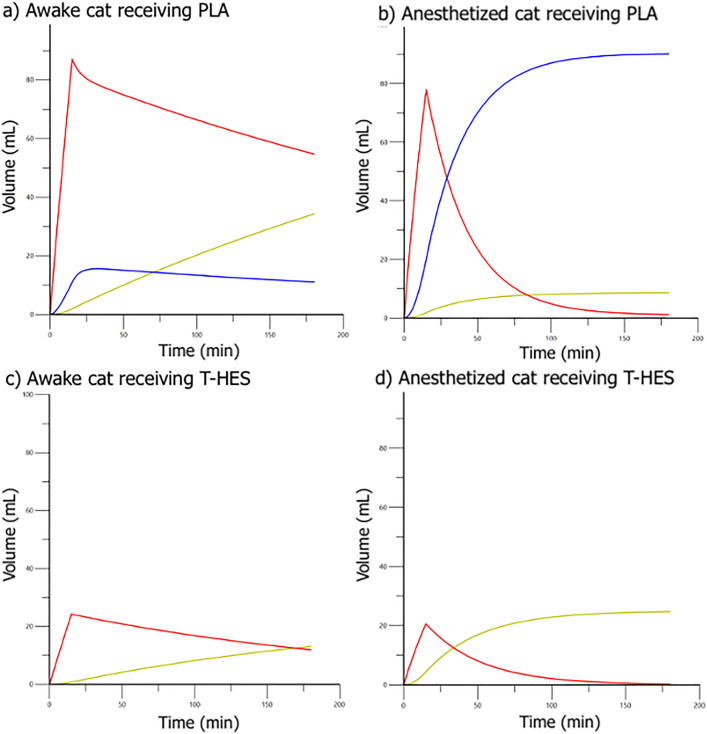
Simulated distribution of 20 mL/kg PLA (a, b) and 5 mL/kg of T-HES (c, d) in conscious (a, c) and anesthetized (b, d) cats into the central compartment (red), peripheral compartment (blue), and elimination (green) as modeled for a 5 kg cat using volume kinetic parameter estimates from Table 3.

**Fig 8:**
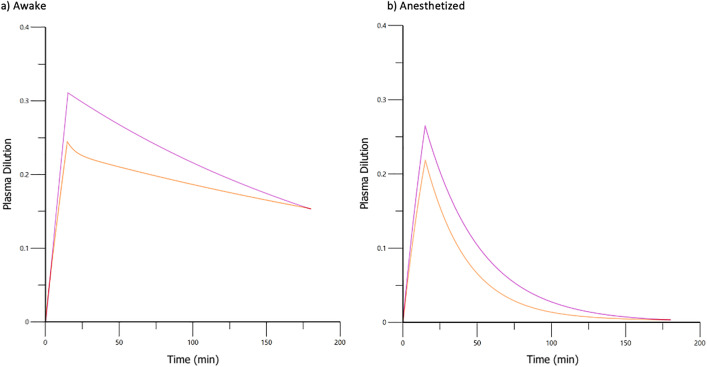
Simulated plasma volume expansion for 20 mL/kg PLA (orange) and 5 mL/kg T-HES (purple) infused over 15 minutes as modeled for a conscious (a) and anesthetized (b) 5 kg cat using volume kinetic parameter estimates from Table 3.

Computer simulation of fluid distribution and plasma volume expansion, using the final VK parameter estimates generated from the combined dataset, indicated a 23–24% plasma volume expansion for PLA and 27–32% for T-HES (Figs 7 and 8).

## Discussion

This study is the first to examine the distribution and elimination of PLA and T-HES in a population of healthy anesthetized cats. All cats completed the study with only minor indwelling central venous catheter complications. VK modeling successfully fitted the combined conscious and anesthetized data sets, producing reasonable VK parameter estimates. General anesthesia, but not MAP, was identified as a significant covariate affecting fluid distribution (decreased *k*_21_ for PLA) and elimination (increased *k*_10_ for T-HES). The slow return of distributed PLA during general anesthesia promoted peripheral edema, and the intravascular persistence of both PLA and T-HES was shorter during general anesthesia than in the awake state.

In our PLA VK analysis, plasma dilution and urine output were successfully fitted to the 2-VOFS kinetic model, where general anesthesia was identified as a significant covariate for decreased *k*_21_ compared to conscious cats. This finding suggests that PLA distributed from the central to the peripheral fluid space returns to the central fluid space at a slower rate in anesthetized cats than in conscious cats. Computer fluid simulation using the final VK parameter estimates from the combined dataset demonstrated that a 20 mL/kg PLA fluid bolus delivered over 15 minutes behaves differently in anesthetized versus conscious cats (Fig 7). In anesthetized cats, administered IV fluids rapidly distribute from the central to peripheral space, with minimal urine excretion over time. Conversely, in conscious cats, more fluid remains in the central space, accompanied by steady urine excretion. Fluid retention within the expandable interstitial space, which is not readily available for renal excretion, helps explain the lower urinary output and anesthesia-associated fluid retention observed in anesthetized cats [11,19,20]. The unidirectional flow of fluid within the lymphatic system is maintained by lymphatic valves, intrinsic contraction of collecting lymphatic vessels, and extrinsic muscle contractions [21]. Anesthetics have been shown to decrease lymphatic flow by suppressing intrinsic lymphatic contractility, while the absence of external forces due to anesthetic-associated muscle relaxation is considered a minor factor [21]. A recent experimental study in mice comparing six different anesthesia protocols demonstrated that isoflurane anesthesia significantly reduced lymphatic contractility, especially in fluid-loaded lymphatic vessels, and delayed the return of lymphatic fluid to the bloodstream [22]. These findings suggest possible mechanisms underlying fluid accumulation in the peripheral space during anesthesia, as observed in our VK model.

A recent study investigated the VK of a balanced crystalloid fluid bolus infused at two different rates (20 mL/kg over 10 or 40 minutes) in healthy anesthetized cats [9]. The study found that *k*_12_ nearly doubled with faster fluid infusion rate, suggesting that rapid bolus infusion increases fluid distribution resulting in a shorter initial volume expansion effect. The area under the curve (AUC) for plasma dilution versus time was not statistically different between the two infusion rates for the first 90 minutes after the bolus, suggesting similar overall volume expansion effects. Both groups exhibited extremely low *k*_21_, indicating fluid retention in the peripheral compartment. Urine output was not measured, which may have impacted the VK model’s stability [25].

Interestingly, general anesthesia, but not MAP, was identified as a significant covariate for PLA retention in the peripheral space (decreased *k*_21_). However, the specific mechanism of general anesthesia responsible for this effect could not be identified. Although the stepwise covariate search identified HR as a covariate, it was not statistically significant and was excluded from the final model. The MAP in the conscious group was significantly different from the anesthetized group as shown in Table 1; however, we did not test our hypothesis over a broad range of MAP (i.e., induced hypotension, hypertension). Thus, if there was a small statistically significant effect of MAP on VK parameters, it could have been missed due to our small sample size. Future studies with larger sample sizes and reduced data variability may provide better insight into the specific components of general anesthesia that is responsible for our findings.

In our T-HES VK analysis, plasma dilution data were successfully fitted to the 1-VOFS kinetic model, where general anesthesia was identified as a significant covariate for increased T-HES elimination (increased *k*_10_) in anesthetized cats. This finding was unexpected, as previous studies have associated general anesthesia with fluid retention [14,23,24]. We speculate that T-HES may have been dissociated into smaller molecules that did not readily cross the vascular endothelium but created a greater osmotic effect. This could have enhanced and sustained volume expansion, ultimately increasing renal blood flow and fluid elimination. However, the specific mechanism by which general anesthesia may have contributed to the plausible increase in T-HES cleavage is unknown. We did not include cumulative urine output as an input variable for T-HES VK analysis. This omission stems from the complexity of T-HES elimination, which is not solely accounted for by urinary excretion. Hydroxyethyl starch consists of large synthetic molecules, and their degradation rate by serum amylase, and consequently their half-life, is determined by molecular weight, molar substitution, and the C2/C6 substitution ratio [25]. Thus, urinary excretion may be a more critical input variable for VK analysis of crystalloids than colloids [26]. In contrast, the model-derived *k*_10_ for T-HES analysis represents the decay of intravascular volume expansion.

Measured urine output during the experimental period was much lower than values reported for conscious cats [8]. The estimated cumulative urine output for conscious cats receiving the same dose of PLA and T-HES as in the current study was approximately 3.5 and 2.5 times greater, respectively, than that observed in anesthetized cats. This finding aligns with previous reports of low urine production and decreased *k*_10_ under anesthesia in VK studies involving humans and sheep [11,23,24,27,28]. The marked decrease in *k*_10_ during anesthesia likely contributes to interstitial edema formation.

The accuracy of oscillometric blood pressure measurement in cats depends significantly on the monitor, with some providing good estimates of MAP in cats [29]. Although direct blood pressure measurement via arterial catheter placement is considered the gold standard, this technique was not chosen for our anesthetized study due to its invasiveness and associated risks [29]. Even if direct measurements had been obtained in our anesthetized cats, the absence of such measurements in the conscious population would have precluded covariate analysis. The modeling software used in this study requires all subjects to have at least one measurement for a parameter to be included as a covariate. Unfortunately, the dataset in healthy conscious cats lacked DBP measurements, thus precluding covariate analysis for DBP.

Visual inspection of the plasma dilution curves for PLA and T-HES in anesthetized cats showed apparent negative plasma dilutions starting around the 60-minute mark (S2 Fig), indicating hypovolemia. Relative hypovolemia due to anesthetic-induced vasodilation is unlikely to account for the observed hypovolemia or affected our VK modeling, as it would not alter blood volume or Hb concentration, which is used to calculate plasma dilution. Similarly, excessive blood loss from sampling or hemorrhage would lower Hb concentration and falsely increase plasma dilution, making it an improbable explanation. Interestingly, no such changes were observed in two other feline VK studies [9,10], contrasting with our findings. One key difference in study design was the use of spontaneous breathing rather than mechanical ventilation. Positive-pressure ventilation is thought to alter circulating blood volume by decreasing venous return and cardiac output, with the magnitude of these effects depending on the patient’s volume status [30]. Thus, the role of positive-pressure ventilation and heart-lung interactions in the observed hypovolemia warrants future investigation.

Based on the revised Starling principle [31], a combination of hydrostatic forces from rapid fluid infusion, endothelial glycocalyx disruption, and the highly compliant feline interstitial matrix [8] may have promoted preferential transvascular fluid efflux into the extravascular interstitial space. Since interstitial fluid primarily returns to circulation via the lymphatic system [31], the suppressive effect of general anesthesia on lymphatic flow may have contributed to fluid accumulation in the interstitium [21,22]. Additional mechanisms that may have contributed to hypovolemia in our study population include evaporative losses through the anesthetic circuit, particularly since no additional fluids were administered after the initial bolus. While evaporative loss in anesthetized cats has not been reported, normal insensible water loss in sedentary conscious cats consuming dry kibble is 29 mL/kg/day [32] and estimated evaporative respiratory water loss is 12 mL/kg/day in high ambient temperatures (41°C) [32]. Under anesthesia with a non-rebreathing circuit, insensible losses are likely higher. Future VK studies may consider constant rate infusion of IV fluids during anesthesia to account for evaporative losses. Additionally, extending the experimental period to measure urinary excretion post-anesthetic recovery may help differentiate between evaporative losses via the anesthetic circuit and fluid ‘trapping’ within the interstitium and lymphatic system.

This study had several other limitations. Although unlikely, it is possible that the concurrent creatinine clearance study may have influenced our results. Creatinine being a non-polar, small (113 Da) molecule and not an effective osmole, is unlikely to significantly influence the reported VK results. To minimize potential confounding effects from the prior bolus injection of creatinine, a 60-minute interval was allowed to ensure equilibrium and distribution before the VK experiment began. Additionally, our study utilized non-splenectomized cats, which may have affected plasma dilution calculations due to RBC sequestration or release. Sympathetic stimulation of splenic contraction is likely less relevant in anesthetized cats [33], but it could be a confounding factor during lighter planes of anesthesia. Since plasma dilution calculations assume that Hb remains constant, splenic contractions leading to increased Hb and RBC measurements could result in a false-negative plasma dilution without actual volume loss. Future VK studies could compare serum albumin dilution with Hb dilution to assess the impact of splenic contractions on the observed hypovolemia. Lastly, our study population consisted of a small homogenous group of young, healthy intact male cats with no surgical trauma. As a result, the findings may not be fully generalizable to the broader clinical patient population. Our relatively small data sample size and possible noisy data may have generated VK parameters that would be difficult to replicate in a different population of cats, especially *k*_21_ in awake state. Thus, readers are encouraged to take away the overarching trend reported in this study rather than using the specific reported VK parameters for clinical decisions.

## Conclusion

Our findings indicate a markedly reduced elimination of IV fluids administered under anesthesia. PLA bolus administration leads to significant fluid retention in the peripheral fluid space and a relatively short plasma expansion effect under anesthesia. Further investigation is warranted to explore the underlying causes of the observed hypovolemia and the potential impact of mechanical ventilation in anesthetized cats.

## Supporting information

S1 File Volume kinetic analysis for healthy anesthetized cats. (DOCX)

S1 Fig Distribution of infused PLA volume into the central compartment (red), second elimination compartment (blue), and urinary excretion (green) as modeled by the modified one-volume of fluid space kinetic model. (TIFF)

S2 Fig Goodness-of-fit plots of observed (open circles) and model-predicted (colored lines) values against time for Plasma-Lyte A (PLA) and 6% tetrastarch 130/0.4 (T-HES) based on a one- volume of fluid space kinetic model in 10 healthy anesthetized cats. (TIFF)

S3 Fig Residual plots from the modified one-volume of fluid space kinetic model of Plasma-Lyte A (PLA) in 10 healthy anesthetized male cats. (TIFF)
