# Supplementary material for: Volume kinetics of crystalloid and colloid solutions administered to healthy anesthetized cats
Source: PLoS One. 2025 Sep 22;20(9):e0333135. doi: 10.1371/journal.pone.0333135 (PMC12453228; doi:10.1371/journal.pone.0333135)
Supplement: S1 File — (DOCX) [file pone.0333135.s001.docx]

**Volume kinetic analysis for healthy anesthetized cats**

Data sets for PLA (E = 10) and T-HES (E = 10) were analyzed separately and both a 1-VOFS and 2-VOFS kinetic model was created. The 2-VOFS model had a lower AIC and -2LL for both fluid types; however, this model was abandoned for PLA due to the failure of model convergence, and HES due to unrealistic parameter estimates with poor confidence (CV% >50%). The 1-VOFS kinetic model was selected for both the PLA and HES based on visual inspection of the plasma dilution curves, rather than a reduction of -2LL. In the 1-VOFS model for PLA, the $k_{10}$ constant in the model predicted a markedly higher elimination compared to the measured urine output. In other words, fluid elimination occurred in the kinetic model at a much higher rate than what was observed in the measured urine output. Thus, fluid was lost from the kinetic system but not observed as urinary excretion. The VK parameter estimates of the PLA and HES models are reported in S1 Table. S1 Fig shows the infused fluid volume represented in plasma expansion, fluid accumulated via the $k_{b}$ elimination rate constant, and urinary excretion over time.

**S1 Table. Volume kinetic parameter estimates from selected models in 10 healthy anesthetized male cats.**

| **Fluid type** | **VK model** | **VK parameter** | **Estimate mean (95% CI)** | **CV (%)** |
| --- | --- | --- | --- | --- |
| PLA  (E = 10) | Modified 1-VOFS^a^ | $V$ | 205 mL^*^ | - |
|  |  | $k_{10}$ | 0.0025 /min (0.0007 – 0.0042) | 35.6 |
|  |  | $k_{b}$ | 0.0282 /min (0.0024 – 0.0540) | 46.4 |
| HES  (E = 10) | 1-VOFS^b^ | $V$ | 60.44 mL (47.88 – 73.00) | 10.5 |
|  |  | $k_{10}$ | 0.0278 /min (0.0197 – 0.0359) | 14.7 |

^a^ Modified 1-VOFS kinetic model with second elimination compartment governed by $k_{b}$.

^b^ 1-VOFS kinetic model with no urinary excretion input.

^*^ Confidence intervals not available as the model failed to converge and required V to be fixed at 205 mL (estimated feline plasma volume) to be stable.

E, number of experiments; PLA, Plasma-Lyte A; T-HES, 6% tetrastarch 130/0.4; VK, volume kinetics; 1-VOFS, one-volume of fluid space; 𝑉, volume of expandable body fluid space, $k_{10}$, first-order elimination rate constant; $k_{b}$, zero-order elimination rate constant CI, confidence interval; CV, coefficient of variation

**S1 Fig. Distribution of infused PLA volume into the central compartment (red), second elimination compartment (blue), and urinary excretion (green) as modeled by the modified one-volume of fluid space kinetic model.**

The differential equation for the modified 1-VOFS kinetic model was:

Modified 1-VOFS kinetic model: $\frac{dv}{dt}=R_{0}-k_{b}- k_{10}(v-V)$

Based on plasma dilution curves, there was a developing trend of hypovolemia over time, as depicted by the increase in negative plasma dilution starting after the 60-minute time point (S2 Fig). As illustrated in the residual plots (S3 Fig), the model-predicted urine output did not fit well with measured urine output for PLA despite additional modeling attempts using a modified 1-VOFS with a new elimination compartment. This was likely a result of the combination of the unstable model fit for the PLA dataset and negative plasma dilution (1).

**S2 Fig. Goodness-of-fit plots of observed (open circles) and model-predicted (colored lines) values against time for Plasma-Lyte A (PLA) and 6% tetrastarch 130/0.4 (T-HES) based on a one- volume of fluid space kinetic model in 10 healthy anesthetized cats.**

**S3 Fig. Residual plots from the modified one-volume of fluid space kinetic model of Plasma-Lyte A (PLA) in 10 healthy anesthetized male cats.**

When we attempted to model the data obtained from the group of anesthetized cats, we encountered several methodological challenges. First, in our attempts to fit either the 1-VOFS or 2-VOFS models for PLA, the 1-VOFS kinetic model was considered appropriate. This finding was unexpected since crystalloid solutions were generally fitted to a 2-VOFS model in the previous feline VK study (2) and previous VK studies in healthy anesthetized humans and experimental sheep (3,4). However, a 1-VOFS model may be selected when justified based on visual evaluation of plotted data and statistical assessments of the model (5). This has been observed in conscious, euhydrated human volunteers where infusion fluids are quickly eliminated from the body (5).

In addition, the urine output predicted by this 1-VOFS model did not agree with the actual output, necessitating the creation of an additional isolated fluid space to attempt to improve the model fit. In an ovine study where healthy splenectomized conscious and anesthetized sheep received 25 mL/kg of normal saline over 20 minutes, similar adaptions of VK analysis with $k_{b}$ representing fluid loss from the kinetic system in a 2-VOFS clearance model were performed (4). The adapted model yielded good curve fits, and model simulation showed very minimal fluid shift by $k_{b}$ (0.0 to 0.1 mL/min) in the conscious groups, compared to a significant fluid loss from the kinetic system in the anesthetized groups (3.2 to 4.2 mL/min). The study concluded that the infused fluid does not distribute only to $V_{p}$, but rather it is lost from the kinetic system to a ‘third space,’ which is functionally isolated from $V_{c}$ and $V_{p}$. A later study with human patients undergoing thyroid surgery had similar findings of ‘third space’ fluid loss ($k_{b}$ = 2.0 mL/min), although to a much smaller magnitude than in the sheep (3). In our study, it is possible that ‘third space’ fluid loss occurred quickly enough that the plasma dilution curves resembled a mono-exponential decline, similar to that observed with crystalloid infusion in conscious humans with fast elimination, resulting in a 1-VOFS model. The distribution of fluid into the non-functional ‘third space’ implies that a fraction of the infused fluid is ‘trapped’ in a compartment that is not available for excretion and is functionally separate from the central and peripheral fluid spaces. This may provide a possible physiologic explanation for the reportedly high risk of fluid overload in cats (6).

Interestingly, the fluid simulation of 20 mL/kg PLA over 15 minutes for an anesthetized cat (Fig 6b) has an almost identical profile as S1 Fig. This suggests that despite different VOFS models, the modified 1-VOFS model from the anesthetized PLA analysis is similar to the 2-VOFS model from the combined PLA analysis. A recent paper investigated whether crystalloid fluid distributes from the central fluid space to peripheral fluid space at different rates, creating two peripheral compartments and whether these compartments are linked in parallel versus series in awake and anesthetized humans (7). The study suggested that crystalloid infusion was best modelled by a 3-VOFS model arranges in series. The study also found that general anesthesia significantly affected fluid kinetics (lower $k_{10}$ and $k_{21}$, and higher $k_{12}$, which promotes fluid distribution into the peripheral fluid space. It is possible that the 3-VOFS model arranged in parallel is analogous to our modified 1-VOFS model in anesthetized cats, explaining its similarities noted with the computer fluid simulation of anesthetized cats from 2-VOFS model in the combined analysis.

**References**

1. Lee JH, Choo YJ, Lee YH, Rhim JH, Lee SH, Choi BM, et al. Population-based volume kinetics of Ringer’s lactate solution in patients undergoing open gastrectomy. Acta Pharmacol Sin. 2019 May;40(5):710–6.

2. Hahn RG, Warner DS. Volume kinetics for infusion fluids. Anesthesiol. 2010 Aug 1;113(2):470–81.

3. Ewaldsson CA, Hahn RG. Kinetics and extravascular retention of acetated Ringer’s solution during isoflurane or propofol anesthesia for thyroid surgery. Anesthesiol. 2005 Sep 1;103(3):460–9.

4. Connolly CM, Kramer GC, Hahn RG, Chaisson NF, Svensén CH, Kirschner RA, et al. Isoflurane but not mechanical ventilation promotes extravascular fluid accumulation during crystalloid volume loading. Anesthesiol. 2003 Mar 1;98(3):670–81.

5. Hahn RG, Drobin D, Ståhle L. Volume kinetics of Ringer’s solution in female volunteers. Br J Anaesth. 1997 Feb;78(2):144–8.

6. Brodbelt DC, Pfeiffer DU, Young LE, Wood JLN. Risk factors for anaesthetic-related death in cats: results from the confidential enquiry into perioperative small animal fatalities (CEPSAF). Br J Anaesth. 2007 Nov;99(5):617–23.

7. Hahn RG. Evidence of serial connection between the plasma volume and two interstitial fluid compartments. Microvascular Research. 2024 Jan;151:104599.
