# Supplementary figures and images for: Volume kinetics of crystalloid and colloid solutions administered to healthy anesthetized cats
Source: PLoS One. 2025 Sep 22;20(9):e0333135. doi: 10.1371/journal.pone.0333135 (PMC12453228; doi:10.1371/journal.pone.0333135)

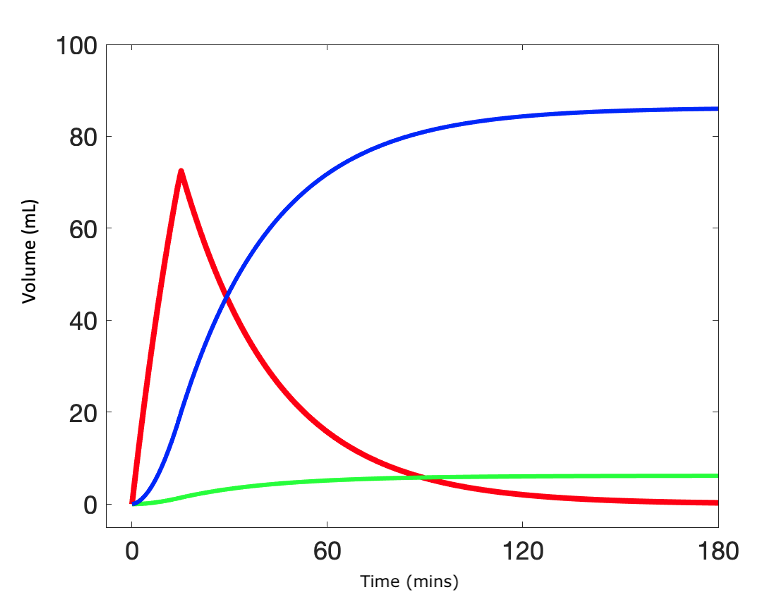

Supplement: S1 Fig — (TIFF) [file pone.0333135.s002.tiff]

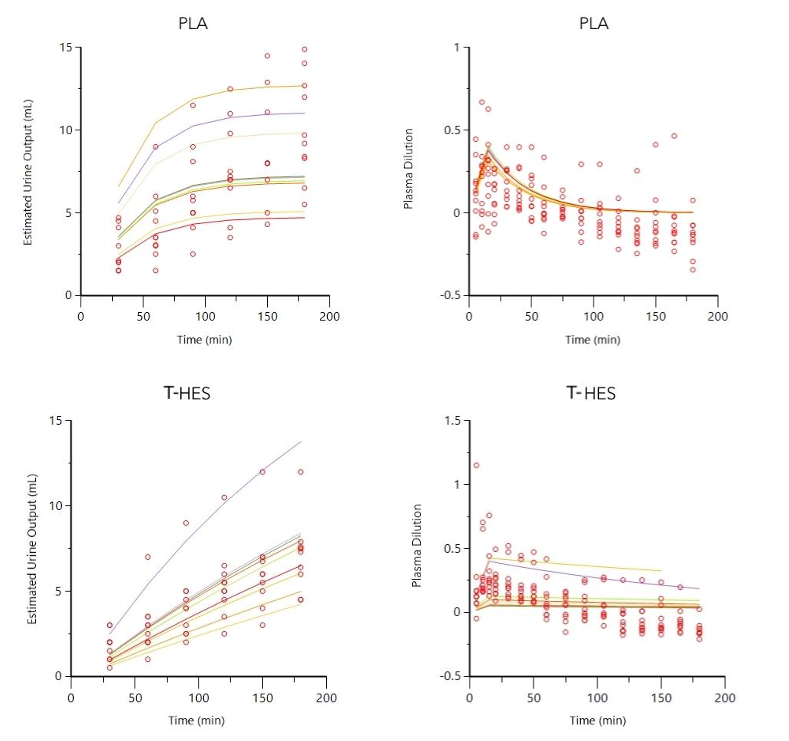

Supplement: S2 Fig — (TIFF) [file pone.0333135.s003.tiff]

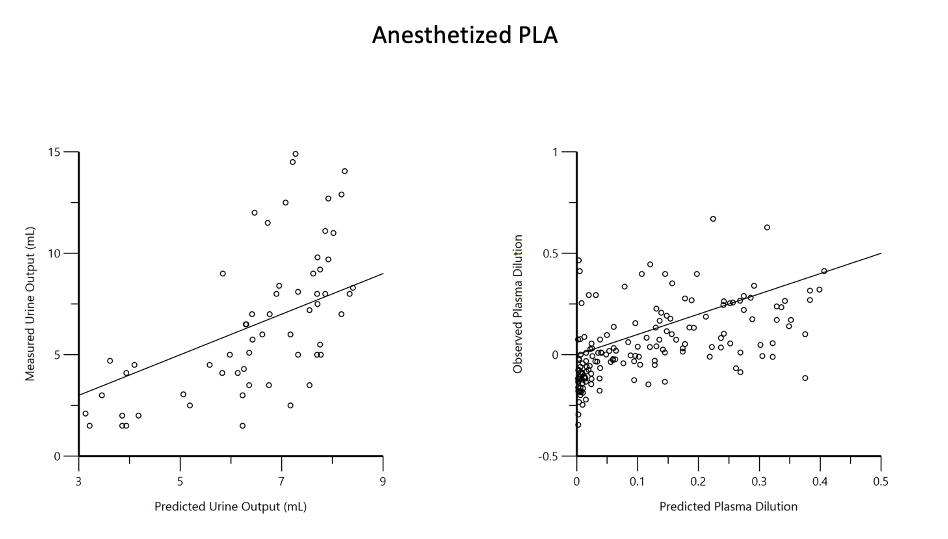

Supplement: S3 Fig — (TIFF) [file pone.0333135.s004.tiff]
